# Comparison of Results from Different Imputation Techniques for Missing Data from an Anti-Obesity Drug Trial

**DOI:** 10.1371/journal.pone.0111964

**Published:** 2014-11-19

**Authors:** Anders W. Jørgensen, Lars H. Lundstrøm, Jørn Wetterslev, Arne Astrup, Peter C. Gøtzsche

**Affiliations:** 1 The Nordic Cochrane Centre, Dept 7811, Rigshospitalet, Copenhagen, Denmark; 2 Copenhagen Trial Unit, Copenhagen Centre of Clinical Intervention Research, Dept 7812, Rigshospitalet, Copenhagen, Denmark; 3 Department of Nutrition, Exercise and Sports, Faculty of Science, University of Copenhagen, Frederiksberg, Denmark; 4 Institute of Medicine and Surgery, Faculty of Health Sciences, University of Copenhagen, Copenhagen, Denmark; University of Ottawa, Canada

## Abstract

**Background:**

In randomised trials of medical interventions, the most reliable analysis follows the intention-to-treat (ITT) principle. However, the ITT analysis requires that missing outcome data have to be imputed. Different imputation techniques may give different results and some may lead to bias. In anti-obesity drug trials, many data are usually missing, and the most used imputation method is last observation carried forward (LOCF). LOCF is generally considered conservative, but there are more reliable methods such as multiple imputation (MI).

**Objectives:**

To compare four different methods of handling missing data in a 60-week placebo controlled anti-obesity drug trial on topiramate.

**Methods:**

We compared an analysis of complete cases with datasets where missing body weight measurements had been replaced using three different imputation methods: LOCF, baseline carried forward (BOCF) and MI.

**Results:**

561 participants were randomised. Compared to placebo, there was a significantly greater weight loss with topiramate in all analyses: 9.5 kg (SE 1.17) in the complete case analysis (N = 86), 6.8 kg (SE 0.66) using LOCF (N = 561), 6.4 kg (SE 0.90) using MI (N = 561) and 1.5 kg (SE 0.28) using BOCF (N = 561).

**Conclusions:**

The different imputation methods gave very different results. Contrary to widely stated claims, LOCF did not produce a conservative (i.e., lower) efficacy estimate compared to MI. Also, LOCF had a lower SE than MI.

## Introduction

Attrition has been described as the bane of clinical trials on anti-obesity drugs [1]. In most studies more than one third of the participants have dropped out after one year [2], and in most cases, missing data leads to bias [3]. Missingness can be classified as missing completely at random (MCAR), missing at random (MAR), or missing not at random (MNAR). In case of MCAR missingness is independent of any observed or unobserved data, e.g. a blood sample that is accidentally dropped on the floor. Only when missingness is MCAR, the use of a complete case analysis of the data, obtained exclusively from participants with all data observed, may give an unbiased result. However, in most studies MCAR is not the case [4]. When missingness is MAR, missingness depends on the observed data, e.g. people with the smallest treatment effect quit the trial. Finally, when missingness is MNAR, it also depends on some unobserved data, e.g. people with an unregistered latent depression quit the trial due to mood changes.

One of the most commonly applied methods for handling attrition in obesity research is ‘last observation carried forward’ (LOCF) [2] where a missing weight measurement of a participant at the end of trial is replaced by the participant's last observed value. To assume that one's weight is unchanged after dropping out of a trial seems hard to justify, as participants tend to regain much of their lost weight within a short period of time after having stopped the intervention [5]. Therefore, 'baseline carried forward' (BOCF) has been proposed as a more reliable imputation strategy [6]. However, both BOCF and LOCF overestimate the precision of the effect estimate because the dataset is analysed, after single imputation of the missing data, as if it was a ‘complete’ dataset with no missing data [7]. Also intuitively, as one has doubts about the imputed data, the p-values and confidence interval should be larger than those computed. Therefore, BOCF and LOCF both provide undue certainty of the effect estimate even under MCAR assumptions.

There are more reliable techniques for handling missing data [8], and they can also be used when missingness is MAR. Attention has been drawn to the multiple imputation (MI) technique [9], which for some time has been recommended for handling missing data in obesity trials [2]. The multiple imputation technique is a stepwise procedure. First, based on the observed data, a plausible multivariable distribution for the missing values is estimated and they are being replaced by values randomly drawn from this distribution resulting in a complete dataset. Second, this procedure is repeated multiple times generating multiple datasets. Third, the datasets are then analysed separately producing multiple estimates, and fourth, the multiple estimates are pooled resulting in one single estimate. Compared to LOCF and BOCF, the precision will be more realistically estimated because the uncertainty of the imputed values is taken into account.

We had access to individual patient data from a large randomised three-armed weight loss maintenance trial that compared diet plus topiramate (96 mg or 192 mg) with diet plus placebo (clinicaltrials.gov PRI/TOP-INT-35) [10]. Our primary aim was to analyse the weight change and compare the results by using four different methods for handling missing data. We analysed the dataset of complete cases and datasets where missing weight measurements had been replaced using three different imputation methods LOCF, BOCF and MI. Our second aim was to report the results of the analysis of the primary outcome measure at the time-point specified in the trial protocol. These were not reported in the published paper [10], because of premature trial termination due to low tolerability of the drug.

## Material and Methods

The trial was a randomised weight loss maintenance trial (n = 561) that compared placebo (n = 187) with topiramate 96 mg (n = 190) and 192 mg (n = 184) per day. It was designed to run for a total of 82 weeks; an 8-week non-pharmacological low-calorie diet run-in phase followed by randomisation, a 60-week intervention phase, a 2-week drug tapering period and 12-week follow-up period. The data included assessments of weight from 26 visits plus standard baseline values (age, height, sex, etc.), a variety of blood sample analyses and measures of hip and waist circumferences. Each visit corresponded to a specific number of weeks in the trial. More details about the methodology have been published previously [10]. The authors of the published trial report wanted to reduce the risk of bias due to premature trial termination and therefore chose to analyse only a subset of people who had received at least one dose of study drug, had provided at least one post-baseline efficacy evaluation, and had the opportunity to complete 44 weeks of treatment before the study closedown announcement. They only allowed data collected before the closedown announcement and up to week 44 to be included in the analysis of efficacy. The primary outcome was percent weight change from enrolment to the end of the intervention phase after 60 weeks of treatment, but this has not been published.

### Data

Data was provided by the first author (AA) of a previous report of this trial [10] and imported to SPSS 18.0.

### Missingness

We assessed the mechanism of missingness by using Little's test [11] and by plotting mean weights of people with missing data with those with data. Little's test is essentially a Chi-square test on whether the complete cases actually consist of a sample chosen completely at random (MCAR) from the intention-to-treat population considering the variables measured on the patients with missingness. P<0.05 excludes a scenario due to MCAR and makes the scenario missing at random (MAR, i.e. missingness is explained by measured variables) more likely, although a scenario of missing not at random (MNAR, i.e. missingness depending on some unobserved variable), can never be totally excluded.

### Comparison of imputation methods

We used the data from baseline (randomisation, week 0) to end of treatment (week 60). For simplicity, we pooled the topiramate arms. We analysed the mean weight change in the placebo and the pooled topiramate group and the difference between the two from baseline to end of treatment. We also analysed percentage change in the same way. The results were plotted against time for comparisons between the four analysis methods. We used the t-test to calculate p-values.

### Complete case analysis

This did not involve any imputation and was an analysis of data from participants on intervention, i.e. all available data from baseline to end of treatment.

### Last observation carried forward

We substituted missing weight measurements with the last observed measurement. We allowed carrying forward the baseline value if this was the last observed measurement.

### Baseline carried forward

We substituted missing weight measurements with the baseline weight.

### Mutiple imputation

We imputed the missing weight measurements with values of weight obtained by the ‘Fully conditional specification method’ in SPSS ver. 18. This is an iterative Markov chain Monte Carlo method that can be used when the pattern of missing data is monotone (i.e. a subject attends all visits till a visit is missed and never returns) or none-monotone [12]. We used a linear regression model that contained the variables: intervention group, sex, race, age and baseline values (height, waist circumference, plasma glucose, triglycerides, HDL-cholesterol, HDL/LDL-ratio, insulin, haemoglobin and haemoglobin A1c). Additionally, we included body weight, but only at visits prior to the visit of interest. For example, we only included weight measurements from baseline to week 20 for the imputation at week 20. We log-transformed weight to satisfy the normality assumption which seemed to hold when we assessed Q-Q plots and used the Kolmogorov–Smirnov test (p>0.2, df = 561), but not the Shapiro–Wilk test (p = 0.04, df = 561). We also assessed convergence by plotting the means and standard deviations by iteration and imputation. The default number of 10 iterations in SPSS 18 seemed to be too low (File S1) as the standard deviation gradually increased up till and stabilized at 400 iterations. Therefore we chose 500 iterations and 10 imputations assuring an efficiency of the imputation of 99%.

### The primary outcome measure at 60 weeks in the trial protocol

We estimated the mean percent weight change from enrolment (week –8) to end of treatment using the same methods as described above. For completeness, we also re-analysed the data on the subset of people previously published [10]). Using the criteria above, it was not possible to get the exact same subset, but when we allowed data collected 3 days after the closedown announcement to be included in the analysis we came close.

## Results

Details about the study population have previously been published [10]. Baseline characteristics are available in File S1 and include the variables used in our analyses after MI.

### Missingness

**Figure 1:**
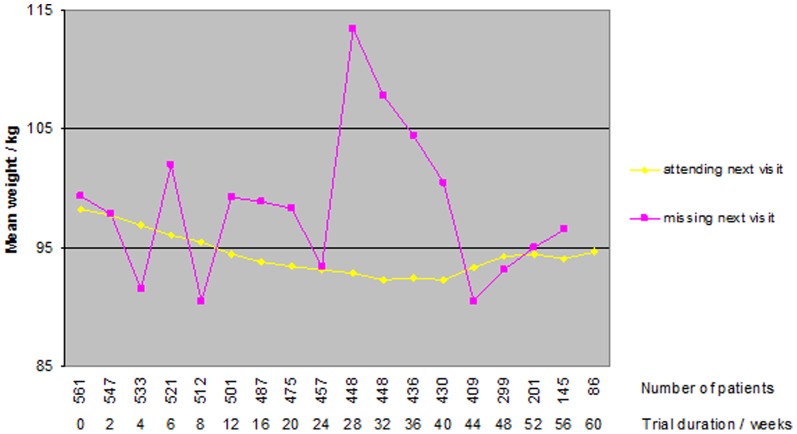
Mean weight of participants attending or missing the next visit. Some patients return after a missed visit. Therefore no change in number of patients at week 28 and 32.

Missing data on weight gradually increased from week 0 to 44 (from 0% to 27%), and then increased markedly (Figure 1); only 15% (n = 86) of the participants were still on treatment and had their weight recorded at the end of treatment. The reason for missingness varied, but although it was mostly caused by premature trial termination [10], the mechanism was unlikely to be MCAR (P<0.01; Little's test)(11). At most visits, participants who missed the following visit seemed to weigh more than those who attended (Figure 1).

### Comparison of imputation methods over time and with increasing missingness

**Figure 2:**
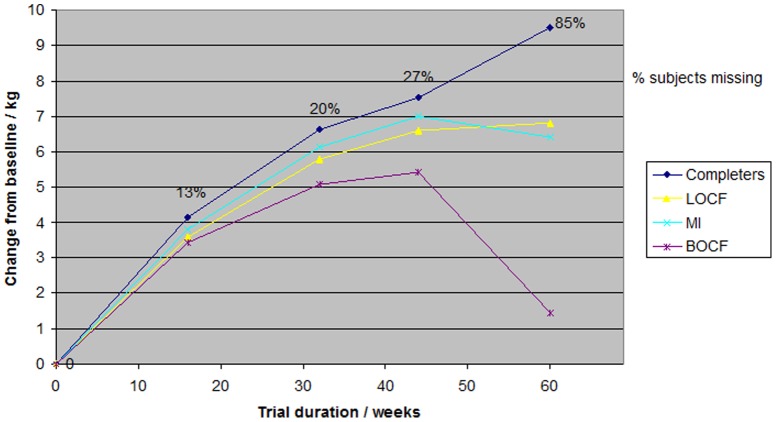
Analysis of difference in weight loss between placebo and topiramate pooled (96 and 192 mg/day) from baseline to week 60 using different methods.

From baseline to week 44, the estimated difference in mean weight change between placebo and topiramate increased by all four methods. From week 44 to end of treatment, the difference increased in the complete case analysis and in LOCF, but decreased in MI and BOCF (Figure 2). From baseline to end of treatment the complete case analysis estimated the greatest difference in weight loss (9.5 kg) and BOCF the smallest (1.5 kg). MI and LOCF were similar with MI resulting in a slightly greater difference from the beginning and throughout most of the trial and a slightly smaller difference at the end of treatment (6.4 kg) compared to LOCF (6.8 kg).

**Figure 3:**
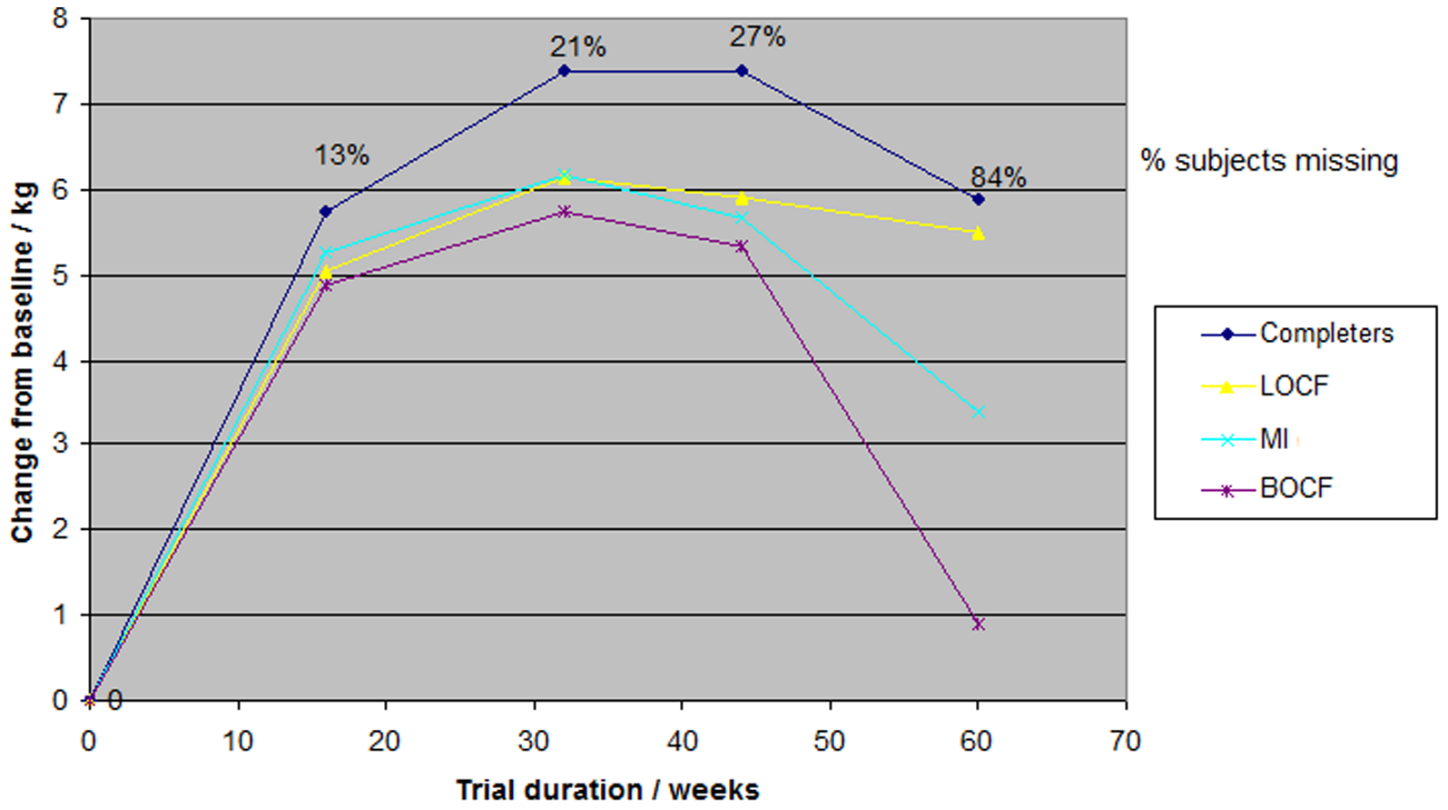
Analysis of weight loss over time in topiramate pooled (96 or 192 mg/day) group using different imputation methods.

**Figure 4:**
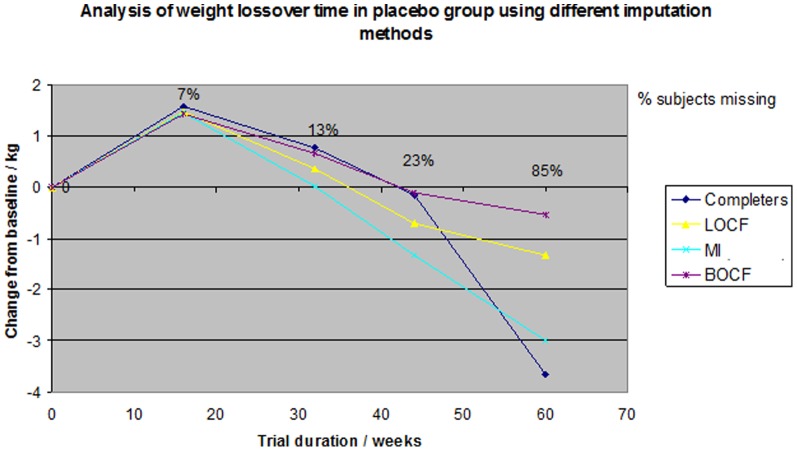
Analysis of weight loss over time in the placebo group using different imputation methods.

These differences were a result of an overall weight loss in the topiramate group (Figure 3) and weight gain in the placebo group (Figure 4). At the end of treatment the weight loss within the pooled topiramate group was again biggest in the complete case analysis (5.9 kg) and smallest in the BOCF (0.9 kg). Also, MI and LOCF estimated a similar weight loss in the beginning of the trial, but at the end of treatment MI (3.4 kg) showed a much smaller weight loss than LOCF (5.5 kg) (Figure 3). The change within the placebo group was similar in the complete case analysis and BOCF in the beginning, but at the end of treatment the complete case analysis estimated the biggest weight gain (3.7 kg) and BOCF the smallest (0.5 kg). In the placebo group, MI and LOCF were similar in the beginning, but at the end of treatment MI (3.0 kg) showed a greater weight gain than LOCF (1.3 kg at week 60) (Figure 4).

We got similar results when we analysed the percentage change from baseline (data not shown).

### The trial's primary outcome measure at 60 weeks

The primary outcome measure at end of trial according to the trial protocol was the percentage change from enrolment (week –8) to end of treatment (week 60), and for placebo compared to topiramate 96 mg the mean difference in weight loss was 10.5% (SE  = 2.2%) in the complete case analysis, 6.1% (SE  = 0.7%) using LOCF, 5.5% (SE  = 1.1%) using MI and 1.7% (SE  = 0.4%) using BOCF. For placebo compared to topiramate 192 mg/day, the mean difference was 10.0% (SE  = 1.9%), 7.5% (SE  = 0.8%), 7.3% (SE  = 1.0%) and 1.5% (SE  = 0.4%) using complete case analysis, LOCF, MI and BOCF, respectively (Table 1). To check the robustness of the findings of this analysis, we also pooled the topiramate groups in a sensitivity analysis and the results were similar (File S1).

**Table 1:** Percentage weight change from enrolment (- 8 week) to end of treatment (week 60).

	Placebo Mean (SE)	Topimarate 96 mg Mean (SE)	Topimarate 192 mg Mean (SE)
Completers	−7.1 (1.40) (n = 28)	−16.2 (1.58) (n = 31)	−16.1 (1.48) (n = 27)
LOCF	−9.2 (0.54) (n = 187)	−14.7 (0.52) (n = 190)	−16.2 (0.65) (n = 184)
MI	−7.7 (0.68) (n = 187)	−12.6 (0.75) (n = 190)	−14.6 (0.76) (n = 184)
BOCF	−9.8 (0.26) (n = 187)	−11.3 (0.33) (n = 190)	−11.5 (0.30) (n = 184)

SE: standard error.

For each difference (topiramate - placebo), P<0.001 (t-test).

When we re-analysed the percentage change from enrolment to week 44 of the subset of participants, our results were similar to those published previously (File S1).

## Discussion

We compared 4 methods of analysing the effect of topiramate in a weight loss trial. We found that, in the beginning of the trial, LOCF and MI estimated similar body weight changes, but over time and with high attrition they estimated different changes. This, however, did not have a substantial impact on the difference in body weight change between topiramate and placebo, which was similar throughout the trial.

Complete case analysis estimated the greatest difference and BOCF the smallest. We also estimated the weight change as originally planned in the trial protocol as it was done in a previous publication of the trial [10], and our results confirmed, regardless of method, that in this trial topiramate produces a greater weight loss than placebo. Other placebo-controlled trials have also shown that topiramate reduces weight [13].

In several simulation studies, MI has been shown to provide a more accurate and broader confidence interval and a less biased estimate of the intervention effect than both complete case analysis and single imputations [14] [15] [16]. In these studies, the method has been to simulate missingness based on a complete dataset and to use different imputation techniques to calculate an estimate of the intervention effect from the imputed dataset. Thus, these studies have been able to evaluate how close the result of an imputation method comes to the correct estimate from the original complete dataset. The results of such simulation studies have been overwhelmingly in favour of MI.

In trials on anti-obesity drugs, it has previously been shown that the LOCF estimates similar effect sizes, but overestimates the precision, compared to MI [2] [8]. It is therefore wrong to describe LOCF as a conservative analysis, although this is often done.

Our results can be interpreted in relation to the *per-protocol (PP) assumption* that all participants adhere to treatment, which is an unrealistic scenario in trials on anti-obesity drugs, or the *intention-to-treat (ITT) method* that assumes that some participants do not adhere to treatment. Thus the PP analysis estimates the weight loss as if the participants adhere to the treatment and the ITT analysis estimates the weight loss of the intention to give the treatment regardless of adherence [17].

If a drug truly reduces the weight, the BOCF will yield a conservative estimate (smaller weight loss) than a PP analysis, but maybe a realistic estimate compared to an ITT analysis [6], as it is likely that participants regain some of their body weight when they stop treatment [5].

The interpretation of the LOCF analyses is difficult due to the course of weight change during a weight loss trial. Most of the weight loss occurs early on, then levels out and some is regained at the end of the trial [18]. Compared with a PP analysis, it may be reasonable to assume that LOCF underestimates the weight loss in the short term and overestimates it in the long term. On the other hand, compared with an ITT analysis, it is likely that LOCF overestimates the weight loss and therefore BOCF should be preferred for LOCF.

Our MI analysis is more compatible with the PP assumption than the ITT assumption [17], because the imputations were based on participants who were on treatment, topiramate or placebo. If we had had complete data on some of the participants that dropped out or did not adhere to the treatment, we could have used their data for MI, which would then have reflected an ITT analysis [17]. When no such data is available the MI analysis is clearly preferable to the biased PP analysis that only includes participants that have adhered to the protocol, but readers and researchers need to think carefully about the analytic assumption (e.g., PP versus ITT) used in an obesity trial.

Theoretically, the complete case analysis *can* occasionally be an unbiased PP analysis, but only when the participants in the analysis can be regarded as a random sample of the study population (when the missing mechanism is MCAR, which is rarely the case [4]). In our dataset, the missing mechanism was not MCAR. It seemed that those who had missing data weighed more than those without and thus the complete case analysis overestimated the weight loss.

Results from weight loss trials are often inadequately reported. The weight change within the treatment groups is stated and the p-value may be the only result reported from the comparison between the groups. Sometimes only the difference in weight change between the groups is stated. We have reported body weight change within the groups and between the groups, but also p-values and CI-intervals. We found that MI and LOCF resulted in similar differences between topiramate and placebo, but that the weight change within the groups was smaller in the MI analysis than in LOCF and this difference increased over time and with increasing missing data. The most likely explanation for this is that LOCF, but not MI, ignores the course of weight change (described above) and that the bias LOCF introduces is more or less the same in both treatment groups. Further, MI introduced greater but more realistic uncertainty of the intervention effect estimate than LOCF and BOCF; analyses using LOCF and BOCF can therefore lead to spuriously significant results.

### Limitations

As we do not know the true effect of topiramate or the body weight of the missing participants, it is impossible to validate our findings against a gold standard. Also, we do not know the exact mechanism of missingness. If the mechanism is MNAR, all imputation methods are likely to be biased. However, MI may provide less biased results in this situation as well [16].

Another limitation is that many data were missing simply because the trial was terminated prematurely, which is not a common reason for attrition in obesity trials. The reason for termination was harms. As we did not include harms in our imputation model, missingness could be related to data that are ‘unobserved’ by the MI procedure. On the other hand, the harms could be correlated to the variables we used; in fact, Figure 1 shows that missingness could be predicted by the weight of the participants. This suggests that people experiencing similar harms are more likely to stay in the trial if they have perceived an effect on their weight.

### Suggestions for improved research

The major reason for missing data when participants quit the treatment is their lack of interest for having their weight measured and for obvious reasons trialists cannot force the patient to show up for a visit. Therefore we need weight loss trials that include incentives for participants to be followed up and other logistic methods for measuring the weight and side effects of those participants who decide to quit treatment.

Most trial protocols specify that investigators shall do their best to have a measurement at the end of the specified period, but usually do not provide incentives or methods. The protocol for the current trial stated that, “Participants withdrawn from the study prior to completion of treatment period will be encouraged […] to attend study visits with assessments equivalent to those performed at Visit 23 [end of treatment]”

The protocol for the RIO-North America trial of rimonabant had a similar statement [19]: “For patients with premature treatment discontinuation or patients considered lost to follow-up, the [case report form] must be filled in up to the last visit performed. The Investigator should make every effort to re-contact and to identify the reason why the patient failed to attend the visit and to determine his/her health status and to retrieve study medication.”

When the RIO-trial was published it was criticised in an editorial for only measuring those participants that completed the trial (53%) rather than all patients that were not lost to follow-up (93%) [18]. It is indeed possible to have a high follow-up as seen in a recently published weight loss trial of free meals and an intensive weight loss program. After 24 months the investigators had weight data on 92% of the study participants [20]. If participants don't attend the last visit, one might also contact them by phone and ask them to use their own scale at home. Despite a trend that self-reported weight is underestimated [22], this strategy may still be reliable when comparing intervention and control groups, because the bias introduced is likely to be similar in both groups.

Selective reporting of favourable analyses also occurs. In a placebo-controlled trial of long-term treatment with sibutramine, the authors only published an unadjusted ITT analysis (LOCF) (n = 464). Compared to placebo the body weight reduction with sibutramine 15 mg was 4.8 kg (p<0.001) and with 10 mg 2.8 kg (p<0.01) [21]. In a trial report of the same study submitted to the Danish Medicines Agency, an adjusted analysis of all available participants (including those who were withdrawn) that had their body weight measured at end of treatment (n = 305), showed smaller weight reductions, 3.0 kg (p<0.001) and 2.1 (p<0.01) kg, respectively.

When imputation is undertaken, more reliable techniques, such as MI, should be used and to make a proper ITT analysis, imputation should also be based on weight data collected after withdrawal In general, MI is considered one of the most reliable methods for imputing missing data. It has been available for more than 20 years and in standard statistical programs for about 10 years. But MI is rarely used in randomised trials and has only recently been proposed for trials on anti-obesity drugs [2]. There has been and still is a steadfast tradition of using LOCF in these trials, but policy is changing. The European Medicines Agency has from 2011 implemented a guideline for handling missing data in clinical trials [23] that the drug industry has to follow. The guideline does not find LOCF, (but BOCF) appropriate for chronic conditions such as obesity where the weight will be expected to return to baseline when the treatment is stopped and it describes MI as a more proper imputation technique. Therefore, we assume that we will see more trials using BOCF and MI in the future. With these changes, drug companies will be more motivated for minimising attrition and missing data because increasing attrition and missing data will result in a treatment effect that approaches zero when BOCF is used.

## Conclusions

The different imputation methods gave different results, but all showed that topiramate reduced weight compared to placebo. In anti-obesity trials, imputation is obligatory due to the amount and type of missing data and because the complete case analysis is biased. However, the ITT analysis using LOCF, which in general is considered a conservative analysis, overestimated the precision. We suggest that post withdrawal weight data must be obtained to make a proper ITT analysis.

## Supporting Information

File S1 Supplementary figures and tables.
(PDF)
