# Supplementary material for: Comparison of Results from Different Imputation Techniques for Missing Data from an Anti-Obesity Drug Trial
Source: PLoS One. 2014 Nov 19;9(11):e111964. doi: 10.1371/journal.pone.0111964 (PMC4237333; doi:10.1371/journal.pone.0111964)
Supplement: File S1 — Supplementary figures and tables. (PDF) [file pone.0111964.s001.pdf]

Web appendix

Web figure 1. Convergence chart showing the mean and standard deviation of the imputed values of log transformed weight measurements at each iteration of five imputations.

The purpose of this plot is to look for patterns in the lines. There should not be any, and these should look suitably “random” which is the case for the mean, but note that the standard deviation increases up to iteration number 450 and then reaches a plateau.

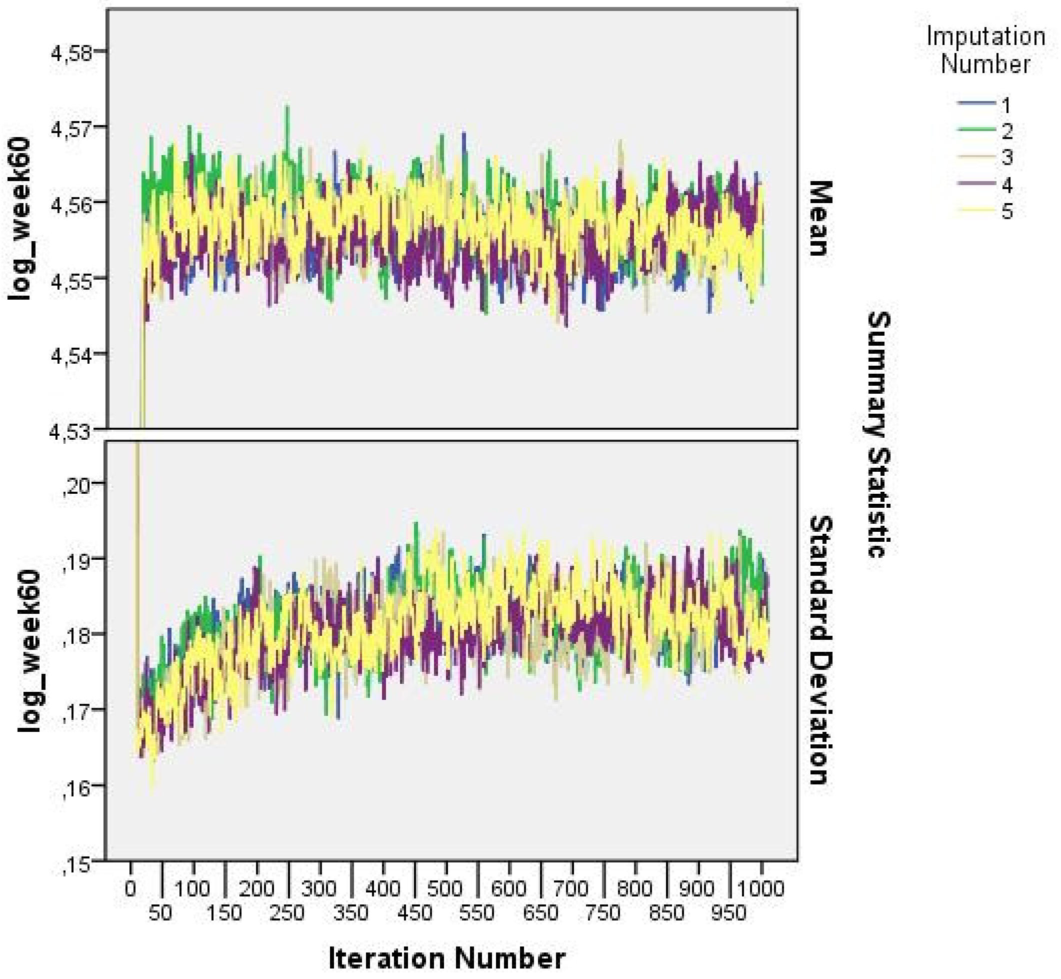

Web table 1. Baseline values for variables included in the model for multiple imputation

	Placebo	Topiramate 96 mg	Topiramate 192 mg	All
Number of participants	187	190	184	561
**Sex**				
Male, no. (%)	39 (21)	53 (28)	44 (24)	136 (24)
Female, no. (%)	148 (79)	137 (72)	140 (76)	425 (76)
**Race**				
White, no. (%)	182 (97)	188 (99)	181 (98)	551 (98)
Black, no. (%)	4 (2)	1 (0.5)	2 (1)	3 (1)
Asian, no. (%)	1 (1)	1 (0.5)	1 (1)	7 (1)
**Mean baseline values**				
Age, years (SD)	44 (11)	44 (10)	44 (11)	44 (11)
Weight, kg (SD)	96.6 (14.7)	98.8 (13.8)	99.3 (15.1)	98.2 (14.5)
Height, cm (SD)	167 (9)	168 (9)	168 (9)	168 (9)
Waist size, cm (SD)	105 (12)	106 (11)	107 (11)	106 (11)
Plasma glucose, mmol/L (SD)*	6.6 (1.8)	6.6 (1.8)	6.7 (1.8)	6.6 (1.8)
Triglycerides, mmol/L (SD)	1.2 (0.5)	1.1 (0.6)	1.3 (0.8)	1.2 (0.6)
HDL cholesterol, mmol/L (SD)	1.2 (0.3)	1.2 (0.3)	1.2 (0.3)	1.2 (0.3)
HDL/LDL cholesterol, ratio, (SD)	2.8 (1.0)	2.9 (1.0)	2.8 (0.9)	2.8 (1.0)
Insulin, mcUI/ml (SD)	10.2 (5.0)	10.7 (8.4)	11.2 (6.2)	10.7 (6.7)
Haemoglobin, g/L (SD)	143 (13)	144 (12)	143 (13)	143.3 (12.3)
Haemoglobin 1Ac, percent (SD)	5.5 (0.5)	5.5 (0.5)	5.5 (0.5)	5.5 (0.5)

*Oral glucose tolerance test

Web table 2. Percentage weight change from enrolment (- 8 week) to week 44 for the subset of participants published in Obes. Res. 2004;12:1658-1669.

	Placebo		Topiramate 96 mg				Topiramate 192 mg			
Analysis	Mean	SE	Mean	SE	CI95%		Mean	SE	CI95%	
					Low	High			Low	High
Completers	(n=83)		(n=71)				(n=81)			
Change, %	-9.6	0.78	-17.0	0.81			-17.6	0,98		
Difference,% (topiramate - placebo)			7.4	1.12	5.1	9.6	8.0	1.3	5.6	10.5
LOCF	(n=99)		(n=96)				(n=105)			
Change, %	-9.1	0.69	-15.3	0.71			-16.6	0.84		
Difference,% (topiramate - placebo)			6.2	0.99	4.2	8.1	7.5	1.10	5.4	9.7
MI	(n=99)		(n=96)				(n=105)			
Change, %	-8.8	0.77	-14.7	0.86			-16.4	0.93		
Difference,% (topiramate - placebo)			5.9	1.15	3.7	8.2	7.6	1.23	5.2	10.0
BOCF	(n=99)		(n=96)				(n=105)			
Change, %	-9.6	0.65	-15.1	0.69			-16.0	0.81		
Difference,% (topiramate - placebo)			5.6	0.95	3.7	7.4	6.4	1.05	4.4	8.5

SE: standard error
CI 95%: 95% confidence interval
Each difference (topiramate - placebo) had a P-value < 0.001 (unpaired t-test)

Web table 3. Sensitivity analysis. Percentage weight change from enrolment (- 8 week) to 60 weeks

	Placebo		Topiramate pooled	
Analysis	Mean	SE	Mean	SE
Completers	(n=28)		(n=58)	
Change, %	-7.1	1.40	-16.2	1.08
LOCF	(n=187)		(n=374)	
Change, %	-9.2	0.54	-15.5	0.42
MI	(n=187)		(n=374)	
Change, %	-7.7	0.68	-13.6	0.53
BOCF	(n=187)		(n=374)	
Change, %	-9.8	0.26	-11.4	0.23

SE: standard error
Each difference (topiramate - placebo) had a P-value < 0.001 (unpaired T-test)
